# Adjuvant effect of herbal medicine on transarterial chemoembolization in patients with hepatocellular carcinoma: A systematic review and meta-analysis

**DOI:** 10.3389/fonc.2023.1106827

**Published:** 2023-02-09

**Authors:** Hyeon-Muk Oh, Eun-Ji Kim, Hye-Ri Bae, Jung-Hyo Cho, Chang-Gue Son, Nam-Hun Lee

**Affiliations:** ^1^Daejeon Korean Medicine Hospital, Daejeon University, Daejeon, Republic of Korea; ^2^East-West Cancer Center, Cheonan Korean Medicine Hospital, Daejeon University, Daejeon, Republic of Korea; ^3^Liver and Immunology Research Center, Daejeon Korean Medicine Hospital of Daejeon University, Daejeon, Republic of Korea

**Keywords:** herbal medicine (HM), transarterial chemoembolization, hepatocellular carcinoma, overall survival (OS), systematic review & meta-analysis

## Abstract

**Objectives:**

Primary hepatocellular carcinoma (HCC) is one of the leading causes of cancer-related deaths, especially in Asian countries. As a practical treatment option, transarterial chemoembolization (TACE) has been well applied; however, its limited efficacy remains challenging. This study analyzed the adjuvant effects of herbal medicine on TACE to determine whether it improves clinical outcomes in patients with HCC.

**Methods:**

A systematic review and meta-analysis was performed to compare the adjuvant effects of herbal medicine on TACE versus TACE therapy alone. We searched the literature from eight databases since January 2011.

**Results:**

Twenty-five studies involving 2,623 participants were selected. The adjuvant therapy of herbal medicine on TACE improved the overall survival at 0.5 years (OR = 1.70; 95% CI 1.21-2.38), 1 year (OR = 2.01; 95% CI 1.65-2.46), 2 years (OR = 1.83; 95% CI 1.20-2.80), and 3 years (OR = 1.90; 95% CI 1.25-2.91). The combination therapy also increased the tumor response rate (OR = 1.84; 95% CI 1.40-2.42).

**Conclusions:**

Despite the unsatisfactory quality of the included studies, the adjuvant therapy of herbal medicine on TACE may provide survival benefits to patients with HCC.

**Systematic reviews registration:**

http://www.crd.york.ac.uk/PROSPERO, identifier (376691).

## 1 Introduction

Hepatocellular carcinoma (HCC) is the leading cause of cancer-related deaths, with an incidence of 9.3 and a mortality rate of 8.5 per 100,000 in 2018 worldwide (1). Since most HCCs are asymptomatic until they reach an advanced or late stage, HCC is difficult to diagnose and has a very poor prognosis (2). The mortality of patients with HCC has remained unchanged over the past decade (3, 4).

Adequate treatments for HCC, including surgical resection, chemotherapy, radiation therapy, radiofrequency ablation, and transarterial chemoembolization (TACE), have improved the 5-year survival rate of HCC from 9% to 18% between 2001 and 2019 (4–7). Among those therapeutics, TACE is the first-line treatment for patients with early-stage and localized HCC and causes tumor necrosis by injection of chemotherapeutic agents into the hepatic artery (8). TACE is not only applied in the early stage but is also frequently used in the unresectable and late stages for palliative care in HCC patients (9).

Approximately 80% of patients with HCC have liver fibrosis, resulting in liver cirrhosis due to chronic inflammation in the liver (10). Although TACE is a topical treatment that can minimize systemic inflammation, TACE accelerates the hepato-fibrotic changes because of its inevitable cytotoxic effects (11). Patients who receive TACE therapy sometimes suffer from complications such as acute cholecystitis, leukopenia, pulmonary embolism, hepatic abscess, bile duct injury, and gastric mucosa injury (12–14). The limitations of TACE in the clinic include not only an insufficient response but also the adverse effects listed above (15).

On the other hand, herbal medicine has been prescribed as an option for patients with hepatic inflammation and liver fibrosis in Asian countries (16, 17). In 1996, the effect of combination therapy of TACE and herbal drugs was first reported (18), and a systematic review of the beneficial outcomes of herbal medicine on TACE was published in 2013 (19). To date, the adjuvant therapy of herbal drugs on TACE for patients with HCC has been further practiced and has been continued; however, no comprehensive evaluation of the combination therapy has been conducted in the last 10 years.

Herein, we conducted a systematic review and meta-analysis to evaluate the adjuvant effect of herbal medicine on TACE in patients with HCC.

## 2 Materials and methods

### 2.1 Protocol and registration

This systematic review, including a meta-analysis, was conducted based on the PRISMA guidelines and was registered in the International Prospective Register of Systematic Reviews (PROSPERO) (ID: 376691, http://www.crd.york.ac.uk/PROSPERO).

### 2.2 Search strategy

Eight databases, including the PubMed, Cochrane, ClinicalTrials.gov, EMBASE, Google Scholar, Chinese National Knowledge Infrastructure, Research Information Sharing Service, and Korean Studies Information Service System databases, were searched after January 2011 using keywords related to primary HCC, herbal medicine, TACE and overall survival. The search terms were (hepatocellular carcinoma OR hepatocellular neoplasms OR liver cancer OR liver neoplasms OR primary hepatic cancer OR intrahepatic neoplasms OR liver adenoma OR liver carcinoma OR hepatocellular adenoma OR HCC) AND (TACE OR transcatheter arterial chemoembolization OR embolization) AND (herb OR herbal medicine OR herbal decoction OR herbal drugs OR phytotherapy OR Korean medicine OR Chinese medicine).

### 2.3 Selection criteria

The studies that met the following criteria were included: clinical studies comparing the effects between ‘TACE combined with herbal drugs’ and ‘TACE-only’ in patients with primary HCC. There was no limit on the language, and studies that did not meet the above criteria were excluded.

### 2.4 Data extraction and review process

After screening the title and abstract of all the studies, the full text of the relevant articles was assessed by two reviewers. Any disagreement was resolved by discussion or consensus with the corresponding author. We conducted a systematic review on the clinical benefits of herbal medicine combined with TACE compared to TACE alone. We extracted the following data: name of the first author, patient information, sample size, herbal medicine, duration of herbal medicine, observation period, and outcome measurements (overall survival at 0.5, 1, 2, and 3 years, number of complete/partial responses, and/or Karnofsky performance status (KPS) score) of the study.

A meta-analysis was performed using odds ratios (ORs) for the overall survival rate and tumor response rate and weighted mean differences (WMDs) for the KPS score with 95% confidence intervals (CIs). Random-effect models were used due to heterogeneity. Dichotomous data are expressed as the OR with 95% CI. WMDs with the 95% CI were calculated for continuous data. The Higgins I^2^ test was used to assess the heterogeneity of the data (20). Statistical significance was set at P < 0.05. Review Manager 5.4.1 was used for the analysis (http://www.tech.cochrane.org/revman) (accessed on 15 July 2022) (21).

## 3 Results

### 3.1 Characteristics of the included studies

**Figure 1:**
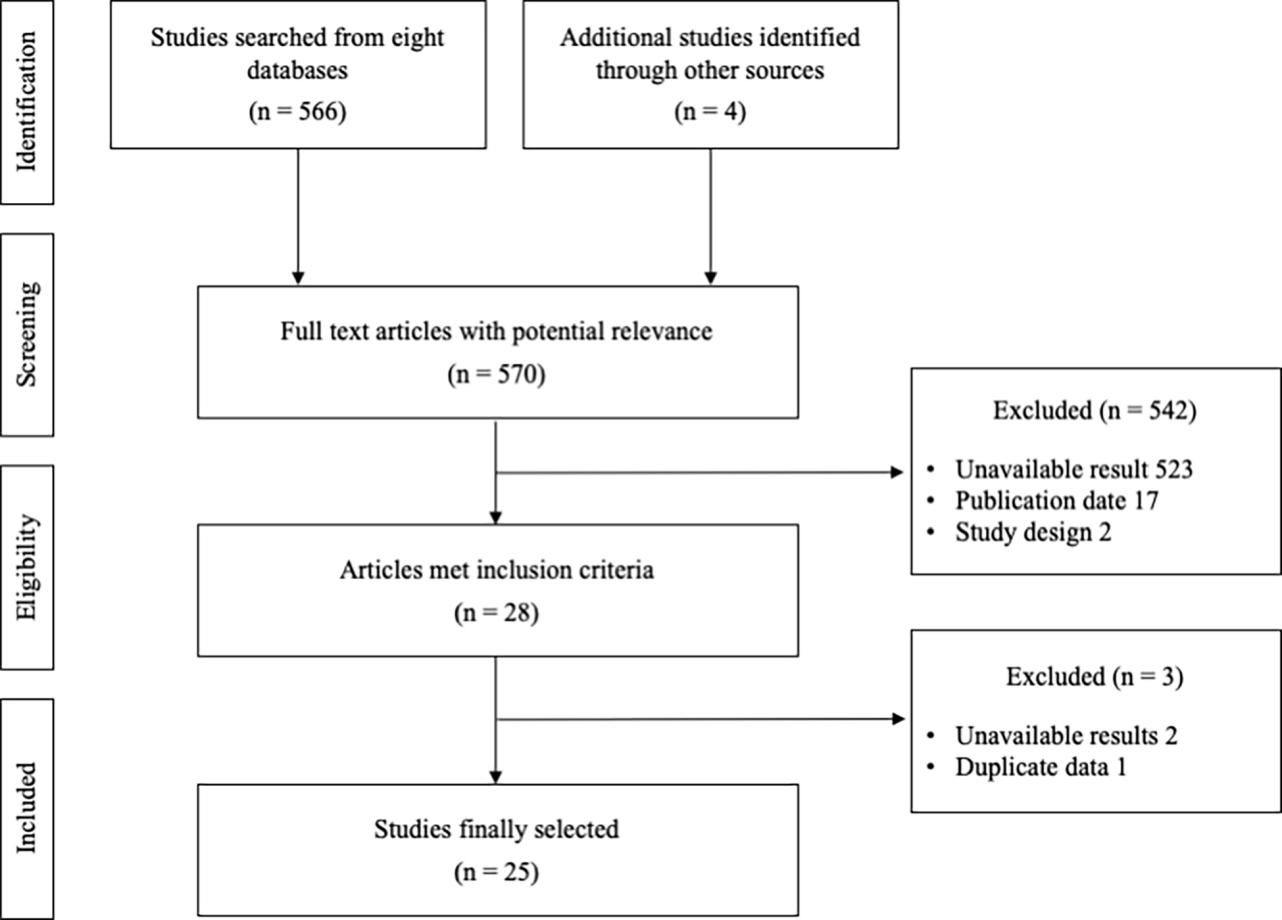
Flow diagram of study selection process.

A total of 570 relevant articles were initially searched, and 25 studies were finally selected for this study (**Figure 1**). The total number of participants was 2,623 (male 1957, female 666), with 1,322 who took the combination therapy and 1,301 who only had TACE. The information for stage was obtained from 655 subjects, and the Child−Pugh scores were determined from 1,746 patients (**Table 1**). There was no significant difference in baseline characteristics between the intervention (TACE+HM) and control (TACE-only), regarding age, sex, HCC stage, Child-Pugh grade, etc. respectively (**Table 1**).

**Table 1:** Basic characteristics of the included studies.

Variable	Intervention (TACE+HM)	Control (TACE-only)	Total
N. of studies			25
N. of participants (%)			
Male	*981	976	1,957 (74.6)
Female	341	325	666 (25.4)
Total	1,322	1,301	2,623 (100.0)
Mean age of participants^*^	53.5 ± 6.7	53.4 ± 5.7	53.4 ± 6.4
HCC stage (N. of participants, %)			
2	136	129	265 (10.1)
3	168	173	341 (13.0)
4	24	25	49 (1.9)
Unknown	994	974	1,968 (75.0)
Child-Pugh score (N. of participants, %)			
A	621	621	1,242 (47.4)
B	241	237	478 (18.2)
C	11	15	26 (1.0)
Unknown	449	428	877 (33.4)
Kinds of herbal medicine			23
Mean treatment period (weeks)			14.0 ± 12.2
Mean observation period (years)			1.84
Outcome measurement (N. of studies)			
Overall survival			25
Tumor response rate			13
Performance status (KPS)			5
Publication year (N. of studies, %)			
2011-2015			12
2016-2021			13
Country (N. of studies, %)			
China			23
USA			2

TACE; transarterial chemoembolization, HM; herbal medicine, HCC; hepatocellular carcinoma, CR; complete response, PR; partial response, KPS; Karnofsky performance score, AFP; α-fetoprotein.

^*^ The mean age was estimated using the presented mean age of each study (from 24 studies).

Twenty-three kinds of herbal medicines were administered for an average of 14.0 ± 12.2 weeks. The mean observation period was 1.84 years, and overall survival was evaluated as the primary measurement at four main time points (0.5, 1, 2, and/or 3 years), along with the tumor response rate and quality of life as secondary measurements (**Table 1**).

### 3.2 Herbal medicine used for combined therapy with TACE

The kinds of herbal medicine and their composition were all provided, as summarized in **Supplementary Table 1**. Yipi Yanggan decoction was applied in three patients, and the rest of the patients had all different kinds (**Table 2**). The most frequently used herbs were *Atractylodes macrocephala* Koidz. (17 times), Wolfiporia extensa (15 times), *Curcuma longa* L. (12 times), *Bupleurum falcatum* L. (12 times), *Astragalus propinquus* Schischk (11 times) (**Supplementary Table 2**).

**Table 2:** Detailed information of included studies.

First author (year)	N. of participants (M/F) HM+T:T-only	TACE type (chemotherapeutic drugs)	Herbal medicine (Chinese)	Duration of herbal medicine	Outcome measurements
Li et al. (2011) (22)	74 (60/14) 38:36	cTACE (5-FU, DDP, THP)	Herbal decoction^*^	8 weeks	Overall survival at 0.5-, 1-year Number of CR, PR
Lu Y. (2011) (23)	66 (52/14) 33:33	cTACE (5-FU, EPI, L-OHP)	Jianpi Jiedu decoction (健脾解毒汤)	12 weeks	Overall survival at 0.5-, 1-year Quality of life (KPS)
Tian et al. (2012) (24)	133 (77/56) 70:63	cTACE (5-FU, ADM, DDP, MMC)	Jinapi Xiaoji decoction (健脾小蓟汤)	8-12 weeks	Overall survival at 1-, 2-, 3-year Number of CR, PR
Zhang et al. (2012) (25)	83 (54/29) 43:40	cTACE (5-FU, DDP, MMC, THP)	Herbal decoction^*^	8 weeks	Overall survival at 1-year Number of CR, PR
Zhou et al. (2012) (26)	59 (36/23) 32:27	cTACE (DDP, GEM)	Herbal decoction^*^	4 weeks	Overall survival at 0.5-, 1-, 2-year Number of CR, PR
Han et al. (2013) (27)	93 (77/16) 47:46	cTACE (EPI, MMC, FUDR)	Fuzheng Jiedu decoction (扶正解毒汤)	12 weeks	Overall survival at 0.5-, 1-, 2-, 3-year Number of CR, PR
Li et al. (2013) (28)	105 (76/29) 43:62	cTACE (5-FU, MMC, THP)	Brucea javanica oil solution (鸦胆子)	8 weeks	Overall survival at 0.5-, 1-, 2-, 3-year
Deng et al. (2014) (29)	80 (51/29) 42:38	cTACE (5-FU, MMC)	Jianpi Yigan decoction (健脾益肝汤)	12 weeks	Overall survival at 0.5-. 1-, 3-year Number of CR, PR Quality of life (KPS)
Lei et al. (2014) (30)	49 (34/15) 32:17	Unknown	Pingwei Xiaoliu decoction (平胃消瘤汤)	12 weeks	Overall survival at 0.5-, 1-, 2-year
Li et al. (2015) (31)	72 (63/9) 36:36	cTACE (5-FU, DDP, EPI, MMC)	Yipi Yanggan decoction (益脾养肝方)	Unknown	Overall survival at 1-year
Wang et al. (2015) (32)	158 (98/60) 78:80	cTACE (5-FU, L-OHP)	Herbal decoction^*^	6 weeks	Overall survival at 0.5-, 1-, 3-year Number of CR, PR
Zhu et al. (2015) (33)	67 (47/20) 35:32	cTACE (5-FU, ADM, CBP, DDP, EPI, MMC)	Taohong Siwu decoction (桃红四物汤)	Unknown	Overall survival at 0.5-, 1-year Number of CR, PR
He et al. (2016) (34)	60 (54/6) 30:30	cTACE (5-FU, EPI, MMC)	Qingre Jiedu mixture (清热解毒汤)	8 weeks	Overall survival at 0.5-, 1-year Number of CR, PR
Kou et al. (2016) (35)	68 (50/18) 34:34	cTACE (5-FU, EPI, DDP)	Bazhen decoction (八珍汤)	8 weeks	Overall survival at 1-, 2-year Quality of life (KPS)
Liu et al. (2016) (36)	106 (68/38) 53:53	Unknown	Yipi Yanggan decoction (益脾养肝方)	60 weeks	Overall survival at 1-, 2-, 3-year
Zhong et al. (2016) (37)	160 (127/33) 80:80	Unknown	Herbal decoction^*^	4-6 weeks	Overall survival at 1-, 3-year Number of CR, PR
Li et al. (2017) (38)	78 (62/16) 40:38	cTACE (5-FU, DDP, THP)	Baoyuan decoction and Xiaoyao powder (保元湯合逍遥散方加減)	15 weeks	Overall survival at 1-, 2-year
Liu et al. (2017) (39)	50 (37/13) 25:25	cTACE (5-FU, CBP, EPI)	Yipi Yanggan decoction (益脾养肝方)	4.5-5.5 weeks	Overall survival at 0.5-, 1-year Number of CR, PR
Pan et al. (2017) (40)	62 (54/8) 31:31	cTACE (CBP, MMC, THP)	Shentao Ruangan tablet (参桃软肝方)	4-48 weeks	Overall survival at 0.5-, 1-year Number of CR, PR
Song et al. (2017) (41)	80 (48/32) 40:40	cTACE (CBP, MMC, THP)	Wenyang Jiedu formula (温阳解毒汤)	12 weeks	Overall survival at 0.5-, 1-, 2-year Number of CR, PR
Wu Mei et al. (2017) (42)	74 (66/8) 37:37	Unknown	Xiaoliu powder (消瘤散)	12 weeks	Overall survival at 0.5-, 1-year Quality of life (KPS) Number of CR, PR
Wu Yunan et al. (2017) (43)	117 (70/47) 62:55	cTACE (EPI, LOB)	Bielong Ruangan decoction (鳖龙软肝汤)	36 weeks	Overall survival at 1-year Number of CR, PR
Xiao et al. (2018) (44)	364 (311/53) 180:184	cTACE (MMC, THP)	Jiedu granule (解毒颗粒)	24 weeks	Overall survival at 1-, 2-, 3-year
Cui et al. (2019) (45)	74 (42/32) 37:37	cTACE (Unknown)	Herbal decoction^*^	8 weeks	Overall survival at 0.5-, 1-year
Yang et al. (2021) (46)	291 (243/48) 144:147	cTACE (DDP, THP)	Fuzheng Jiedu Xiaoji formula (扶正解毒消积方)	12 weeks	Overall survival at 1-year

T; TACE, HM; Herbal medicine, CR; complete response, PR; partial response, AFT; α-fetoprotein, KPS; Karnofsky performance status, 5-FU; 5-Fluouracil, DDP; Cisplatin, THP; Pirarubicin, EPI; Epirubicin, L-OHP; Oxaliplatin, ADM; Doxorubicin, MMC; Mitomycin, GEM; gemcitabine, FUDR; floxuridine, CBP; Carboplatin, LOB; Lobaplatin. ^*^ The case where only the composition was presented without the specific name of the herbal medicine is indicated.

### 3.3 Benefits in overall survival (primary measurement)

**Figure 2:**
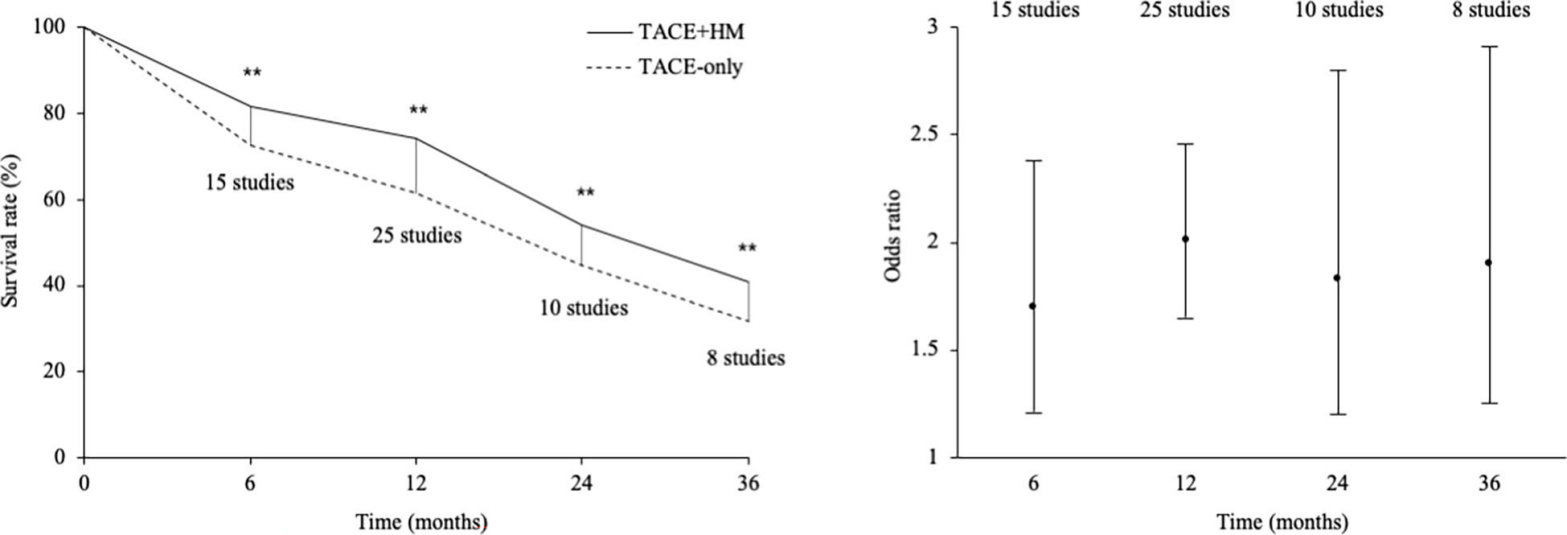
Survival rate and odds ratio at 6, 12, 24, and 36 months. ** indicated that P < 0.05.

**Figure 3:**
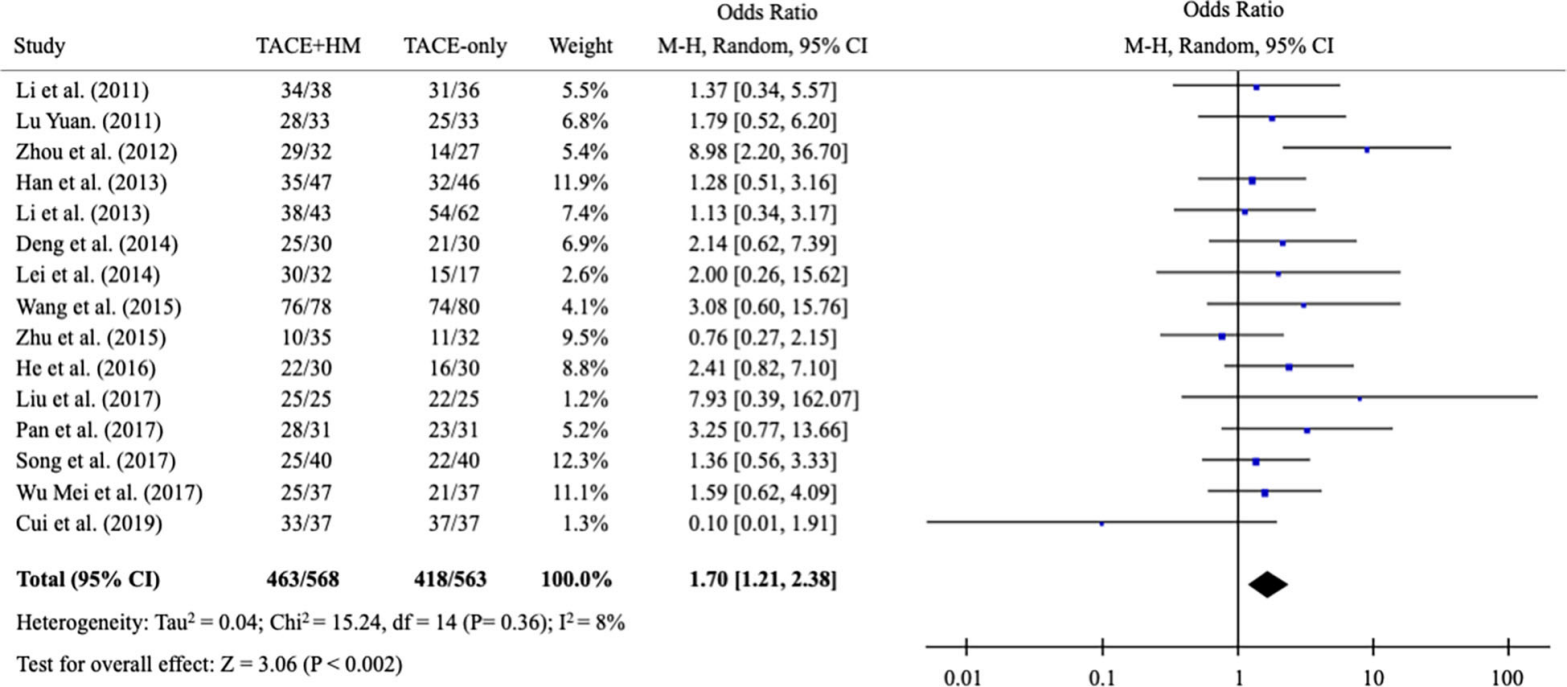
Meta-analysis of overall survival at 6-month.

**Figure 4:**
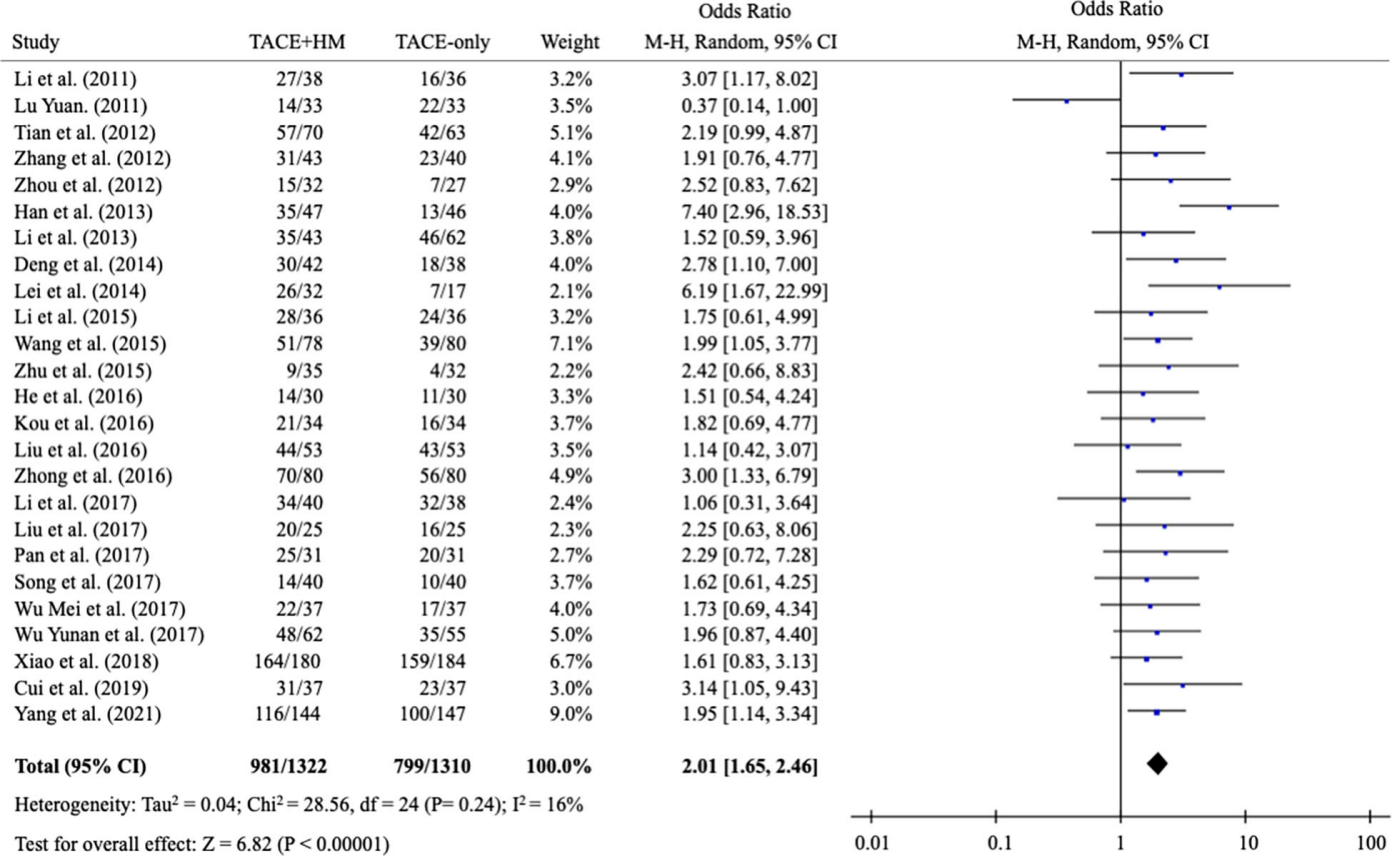
Meta-analysis of overall survival at 12-month.

**Figure 5:**
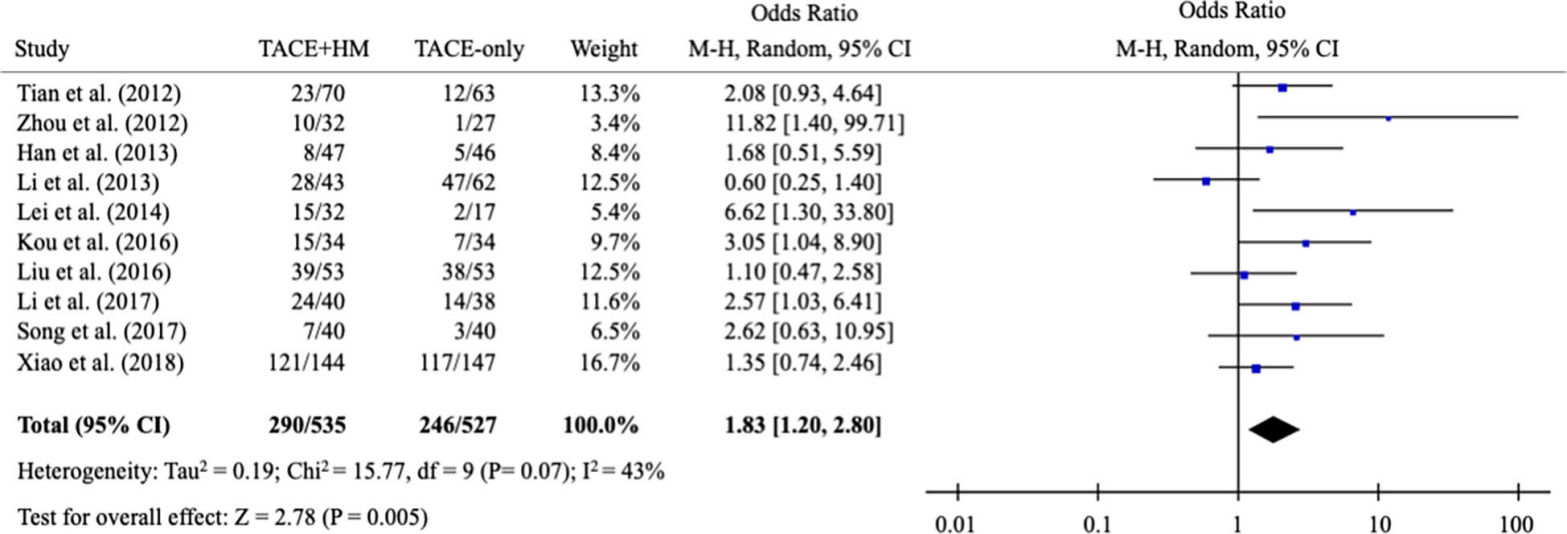
Meta-analysis of overall survival at 24-month.

**Figure 6:**
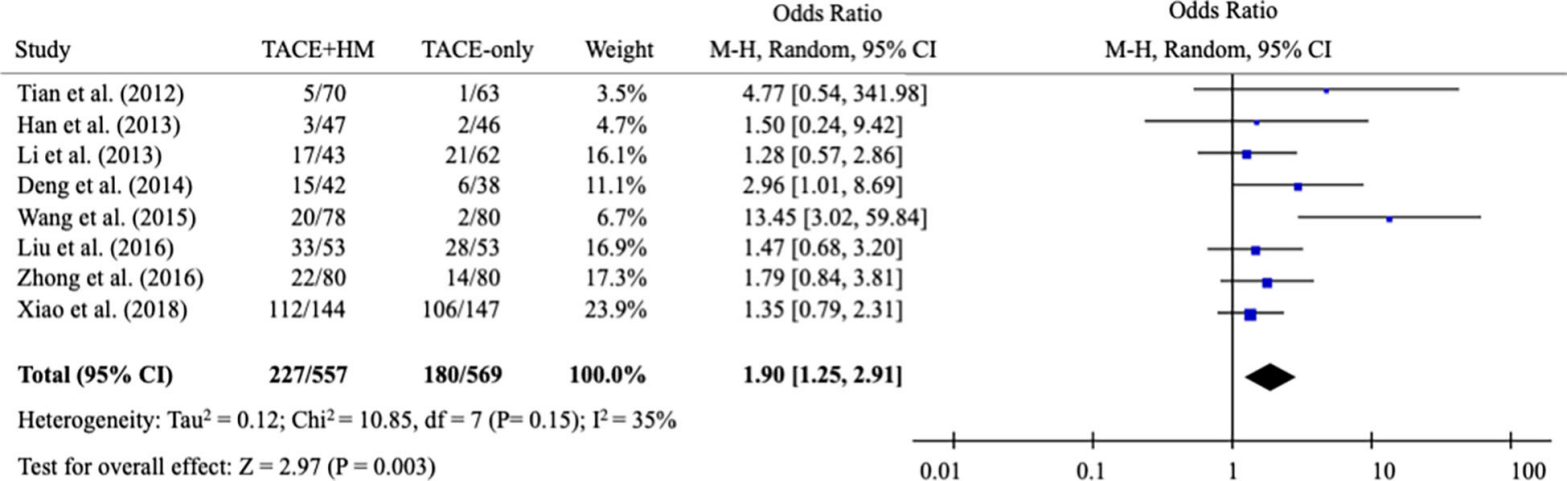
Meta-analysis of overall survival at 36-month.

From the meta-analysis of the overall survival rate, the combination therapy showed a significant improvement in the survival rate at all measured points (**Figure 2**); OR = 1.70 at 0.5 years (95% CI 1.21-2.38; P < 0.002, 15 studies, 1,131 participants) (**Figure 3**), OR = 2.01 at 1 year (95% CI 1.65-2.46; P < 0.00001, 25 studies, 2,623 participants) (**Figure 4**), OR = 1.83 at 2 years (95% CI 1.20-2.80; P = 0.005, 10 studies, 1,062 participants) (**Figure 5**), and OR = 1.90 at 3 years (95% CI 1.25-2.91; P = 0.003, 8 studies, 1,126 participants) (**Figure 6**).

### 3.4 Benefits in tumor response rate and quality of life (secondary measurement)

**Figure 7:**
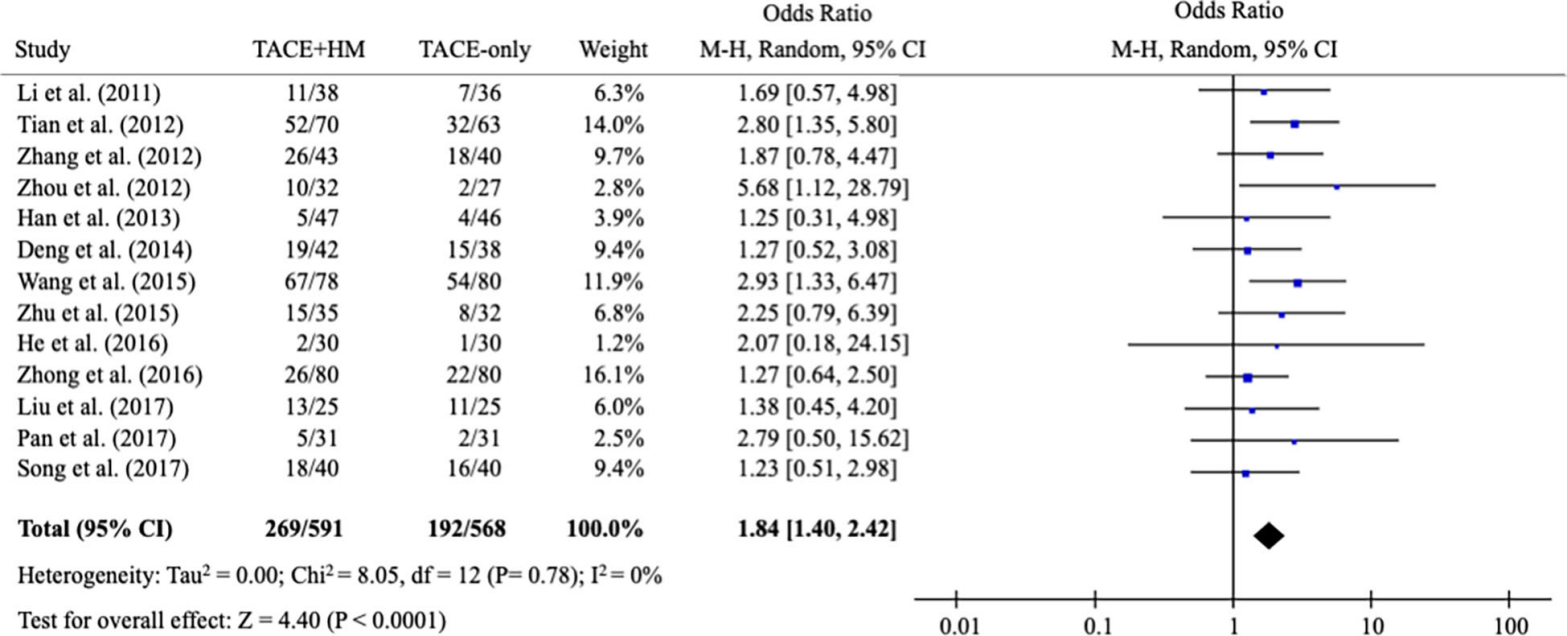
Meta-analysis of the number of complete response/partial response.

As secondary measurements, the meta-analysis for the response rate of treatment was significantly increased in the combination group as the OR = 1.84 (95% CI 1.40-2.42; P < 0.0001) from 13 studies (n=1,159) (**Figure 7**).

**Figure 8:**
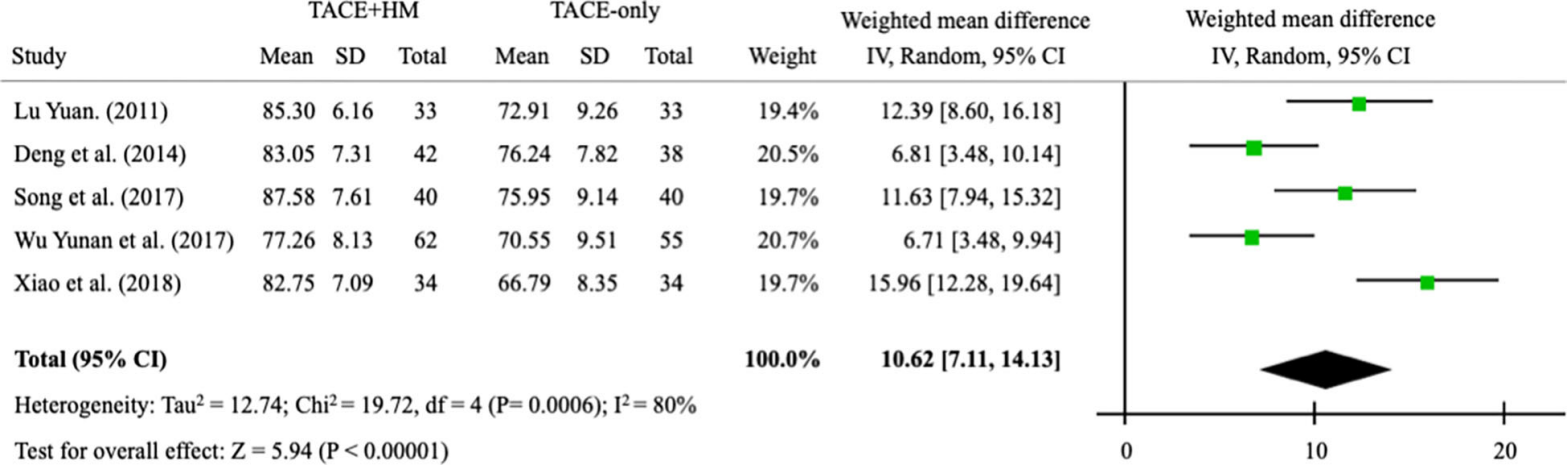
Meta-analysis of performance status (Karnofsky performance status score).

Quality of life measured by the KPS score was significantly improved by combination therapy, with an WMD = 10.62 (95% CI 7.11–14.13; P < 0.00001) from 5 studies (n = 411) (**Figure 8**).

## 4 Discussion

The well-known risk factors for HCC are infection with hepatitis B virus (HBV) or hepatitis C virus (HCV), excessive alcohol consumption, and nonalcoholic fatty liver disease (NAFLD) (47–49). In Asian countries, including China, 70~80% of HCC cases are known to be caused by HBV infections (50), while most of our data for meta-analysis did not describe the causes of HCC. As it is known, there is a male-predominance of HCC patients, and our data showed a 3.0-fold higher number of male patients than female patients (**Table 1**).

From 25 studies containing 2,623 participants (1,322 herbal interventions), we found a 1.29-fold survival benefit compared to the TACE-only group (primary endpoint) (**Figure 2**). The present results may indicate that the add-on therapy on TACE obtained a positive clinical outcome on survival gain. In fact, TACE therapy is usually coadapted with chemotherapies (51). A study reported a 1.6-fold improvement in the survival rate in patients with HCC with Child−Pugh score A using an adjuvant therapy of sorafenib with TACE (52), and that study’s data was slightly superior to our data. In our study, the majority of the participants for whom the stage information was provided (only 25% of the total participants) were stages II and III, and the majority of the patients (from 50% of participants) were Child−Pugh score A. TACE had significant survival advantages compared to supportive care (21.2 vs. 14.5 months for 4 years of observation time) for patients with late-stage HCC (53); thus, the evaluation of the adjuvant effects of herbal medicine are necessary. As for the types of TACE, novel treatment of chemoembolization with drug-eluting beads (DEB-TACE) has been introduced to reduce drawbacks of conventional TACE (c-TACE) and to improve the overall results (54). However, 21 studies except 4 RCTs not described the types of TACE used cTACE in this systematic review (**Table 2**).

Patients with HCC suffer from various symptoms, such as abdominal pain, diarrhea, nausea, vomiting, jaundice, cholangitis, and fever (55). Surgical resection or TACE can cause pain or discomfort and deteriorate the quality of life (56). In our results, the adjuvant treatment of herbal drugs improved the quality of life after treatment by 10.6 out of 100 points compared to the TACE-alone group. This finding is similar to the result of one article that reported quality of life improvements of 10.0 out of 100 points in three-dimensional conformal radiotherapy, which is typically used for adjuvant therapy with TACE (57). Our study supported that herbal medicine could improve the quality of life by relieving symptoms when combined with TACE. On the other hand, hepatic fibrosis is a crucial factor in determining the prognosis of HCC patients, and hepatic fibrosis progresses gradually and leads to fatal outcomes (58). However, there is no optimal therapeutic for liver fibrosis to date (59). Herbal medicines have been investigated as potential treatments for liver fibrosis due to their anti-inflammatory and antiviral properties (60). For example, Chunggan syrup (CGX), a standardized herbal formula in Korea, improved liver fibrosis, as assessed by the decreases in liver stiffness measurement score, in a clinical trial (61). In another trial, oxymatrin, extracted from *Sophora alopecuraides* L., showed a significant antifibrotic effect (with a total effective rate of 48% vs. 4% compared to placebo after 24 weeks of administration) (62). These antifibrotic actions may contribute to survival benefits in patients with HCC treated by TACE. Besides antifibrotic properties, there would be other mechanisms corresponding to adjuvant effects of herbal drugs on TACE, we however currently cannot identify them from present data.

In this review, mostly different kinds of herbal medicine were used in 25 studies, except for in 3 of the patients (**Supplementary Table 1**), and the compositions of these therapies were also diverse (**Supplementary Table 2**). The heterogeneity of the herbal medicines was the main limitation of this study, which makes it difficult to clarify the interaction between herbal medicine and TACE, and their corresponding mechanisms. Other limitations would include the unsatisfactory initial data from relatively poorly designed clinical trials and the possibility of publication bias due to only a very few studies reporting negative outcomes. To strengthen the clinical evidence for the adjuvant efficacy of herbal medicine on TACE therapy to treat HCC patients, further strictly designed clinical trials should be performed that have standardized herbal remedies. Herbal drugs have been adopted worldwide, but concerns regarding their safety have arisen (63). Regarding the adverse effects of combination therapy on HCC, the present data did not show any notable frequency compared to only TACE therapy.

In conclusion, this systematic review and meta-analysis showed survival benefits in patients with HCC by combined treatment with herbal medicine and TACE. The adjuvant effect of herbal drugs on TACE needs to be further evaluated by well-designed RCTs in the future.

## Data availability statement

The raw data supporting the conclusions of this article will be made available by the authors, without undue reservation.

## Author contributions

H-MO: wrote the main manuscript text, and conducted statistical analysis; E-JK, H-RB: contributed to the data collection and manuscript preparation including revision process; J-HC, C-GS: supervised the manuscript; N-HL: supervised the manuscript, and directed final version of all contents. All authors reviewed and approved this manuscript.
