# Supplementary material for: Adjuvant effect of herbal medicine on transarterial chemoembolization in patients with hepatocellular carcinoma: A systematic review and meta-analysis
Source: Front Oncol. 2023 Feb 9;13:1106827. doi: 10.3389/fonc.2023.1106827 (PMC9948036; doi:10.3389/fonc.2023.1106827)
Supplement: Supplementary file 1 [file Table_1.docx]

Supplementary Table 1. Composition of herbal medicine

| First author (year) | Herbal medicine | Composition of herbal medicine |
| --- | --- | --- |
| Li et al. (2011) [37] | Herbal decoction^*^ | *Amomum villosum* Lour., *Angelica gigas* Nakai, *Atractylodes macrocephala* Koidz., *Bupleurum falcatum* L., *Coix lacryma-jobi* L., *Crataegus pinnatifida* Bunge, *Curcuma longa* L., *Cuscuta japonica* Choisy, *Eclipta prostrata* L., Galli Stomachichum Corium, *Inula helenium* L., *Ligustrum japonicum* Thunb., *Melia azedarach* L., *Paeonia × suffruticosa* Andrews, *Paeonia lactiflora* Pall., *Panax ginseng* C.A mey., *Pinellia* Ten., *Psoralea corylifolia* L., *Rehmannia glutinosa* DC., Wolfiporia extensa |
| Lu Y. (2011) [38] | Jianpi Jiedu decoction  (健脾解毒汤) | *Amana edulis* Honda, *Atractylodes macrocephala* Koidz., *Coix lacryma-jobi* L., *Curcuma longa* L., Eupolyphaga sinensis Walker, *Glycyrrhiza uralensis* Fisch. ex DC., *Oldenlandia diffusa* Roxb., *Pseudostellaria heterophylla* Pax, *Scutellaria barbata* D.Don, Wolfiporia extensa |
| Tian et al. (2012) [39] | Jinapi Xiaoji decoction  (健脾小蓟汤) | *Atractylodes macrocephala* Koidz., Olivierus martensii, *Cirtus unshiu* Marcow., *Citri sarcodactylis* Fructus, *Citrus × aurantium* L., *Crataegus pinnatifida* Bunge, *Hordeum vulgare* L., *Oldenlandia diffusa* Roxb., *Oryza sativa* L., *Pinellia* Ten., *Pseudostellaria heterophylla* Pax, Scolopendra subspinipes mutilans L. Koch, *Triticum aestivum* L., Wolfiporia extensa |
| Zhang et al. (2012) [40] | Herbal decoction^*^ | *Artemisia capillaris* Thunb., *Atractylodes macrocephala* Koidz., *Bupleurum falcatum* L., *Codonopsis pilosula* Nannf., *Glycyrrhiza uralensis* Fisch. ex DC., *Paeonia lactiflora* Pall., *Scutellaria barbata* D.Don |
| Zhou et al. (2012) [41] | Herbal decoction^*^ | *Angelica gigas* Nakai, *Atractylodes macrocephala* Koidz., *Bupleurum falcatum* L., *Codonopsis pilosula* Nannf., *Curcuma longa* L., *Dioscorea polystachya* Turcz., *Glycyrrhiza uralensis* Fisch. ex DC., *Inula helenium* L., *Machilus thunbergii* Siebold & Zucc., *Oldenlandia diffus*a Roxb., *Paeonia lactiflora* Pall., Wolfiporia extensa |
| Han et al. (2013) [42] | Fuzheng Jiedu decoction  (扶正解毒汤) | *Actinidia Valvata* Dunn, *Agrimonia pilosa* Ledeb., *Astragalus propinquus* Schischk., *Atractylodes macrocephala* Koidz., *Cirtus unshiu* Marcow., *Crataegus pinnatifida* Bunge, Galli Stomachichum Corium, *Oryza sativa* L., *Salvia japonica* Thunb., *Selaginella tamariscina* Spring |
| Li et al. (2013) [43] | Brucea javanica oil solution  (鸦胆子) | *Acanthopanax senticosus* Harms, *Astragalus propinquus* Schischk., *Brucea javanica* Merr., Mylabris cichorii, *Panax ginsen*g C.A mey. |
| Deng et al. (2014) [44] | Jianpi Yigan decoction  (健脾益肝汤) | *Agrimonia pilosa* Ledeb., *Astragalus propinquus* Schischk., *Atractylodes macrocephala* Koidz., *Bupleurum falcatum* L., *Codonopsis pilosula* Nannf., Galli Stomachichum Corium, *Glycyrrhiza uralensis* Fisch. ex DC., *Ligustrum japonicum* Thunb., *Paeonia lactiflora* Pall., *Salvia miltiorrhiza* Bunge, Wolfiporia extensa |
| Lei et al. (2014) [45] | Pingwei Xiaoliu decoction  (平胃消瘤汤) | *Astragalus propinquus* Schischk., *Atractylodes lancea* DC., *Cirtus unshiu* Marcow., *Codonopsis pilosula* Nannf., *Curcuma longa* L., *Machilus thunbergii* Siebold & Zucc., *Momordica charantia* L., *Oldenlandia diffusa* Roxb., *Panax ginseng* C.A mey., *Phyllostachys bambusoides* Siebold & Zucc., *Pinellia* Ten., *Poncirus trifoliata* Raf., Wolfiporia extensa |
| Li et al. (2015) [46] | Yipi Yanggan decoction  (益脾养肝方) | *Amomum villosum* Lour., *Atractylodes macrocephala* Koidz., *Bupleurum falcatum* L., *Cirtus unshiu* Marcow., *Codonopsis pilosula* Nannf., *Coix lacryma-jobi* L., *Curcuma longa* L., *Dioscorea polystachya* Turcz., *Inula helenium* L., *Levisticum officinale* W.D.J.Koch, *Oldenlandia diffusa* Roxb., *Salvia miltiorrhiza* Bunge, *Scutellaria barbata* D.Don, Wolfiporia extensa |
| Wang et al. (2015) [47] | Herbal decoction^*^ | *Artemisia capillaris* Thunb., *Astragalus propinquus* Schischk., *Atractylodes macrocephala* Koidz., *Bupleurum falcatum* L., *Codonopsis pilosula* Nannf., *Curcuma longa* L., *Gentiana scabra* Bunge, *Inula helenium* L., *Oldenlandia diffusa* Roxb., *Pinellia* Ten., *Prunus persica* Batsch, *Salvia miltiorrhiza* Bunge, *Sparganium stoloniferum* Buch. |
| Zhu et al. (2015) [48] | Taohong Siwu decoction  (桃红四物汤) | Pre-TACE herbal decoction: *Angelica gigas* Nakai, *Albizia julibrissin* Durazz., *Bupleurum falcatum* L., *Carthamus tinctorius* L., *Cirtus unshiu* Marcow., *Citrus × aurantium* L., *Curcuma longa* L., *Dendranthema indicum* Des Moul., *Forsythia Koreana* Nakai, *Glycyrrhiza uralensis* Fisch. ex DC., Galli Stomachichum Corium, *Levisticum officinale* W.D.J.Koch, *Lonicera japonica* thunb., *Paeonia lactiflora* Pall., *Prunus persica* Batsch, *Melia azedarach* L., *Phyllostachys bambusoides* Siebold & Zucc., Wolfiporia extensa  Post-TACE herbal decoction: *Astragalus propinquus* Schischk., *Atractylodes macrocephala* Koidz., *Corydalis* yanhusuo, *Cirtus unshiu* Marcow., *Glycyrrhiza uralensis* Fisch. ex DC., *Forsythia Koreana* Nakai, Ganoderma lucidum, *Nelumbo nucifera* Gaertn., *Paeonia lactiflora* Pall., *Pinellia* Ten., *Plantago lanceolata* L, *Tetradium ruticarpum* T.G.Hartley, Wolfiporia extensa |
| He et al. (2016) [49] | Qingre Jiedu mixture  (清热解毒汤) | *Actinidia Valvata* Dunn, *Agrimonia pilosa* Ledeb., *Astragalus propinquus* Schischk., *Atractylodes macrocephala* Koidz., *Cirtus unshiu* Marcow., *Crataegus pinnatifida* Bunge, *Hordeum vulgare* L., *Oryza sativa* L., *Salvia japonica* Thunb., *Selaginella tamariscina* Spring |
| Kou et al. (2016) [50] | Bazhen decoction  (八珍汤) | *Angelica gigas* Nakai, *Atractylodes macrocephala* Koidz., *Codonopsis pilosula* Nannf., *Glycyrrhiza uralensis* Fisch. ex DC., *Levisticum officinale* W.D.J.Koch, *Paeonia lactiflora* Pall., *Rehmannia glutinosa* DC., Wolfiporia extensa |
| Liu et al. (2016) [51] | Yipi Yanggan decoction  (益脾养肝方) | *Alisma canaliculatum*, *Asparagus cochinchinensis* Merr., *Astragalus propinquus* Schischk., *Carthamus tinctorius* L., *Crataegus pinnatifida* Bunge, *Dioscorea polystachya* Turcz., *Hordeum vulgare* L., *Lycium chinense* Mill., Magnetite, Massa Medicata Fermentata, *Paeonia lactiflora* Pall., *Panax ginseng* C.A mey., Pelodiscus maackii, *Prunus persica* Batsch, *Pseudostellaria heterophylla* Pax, *Trichosanthes kirilowii* Maxim. |
| Zhong et al. (2016) [52] | Herbal decoction^*^ | *Atractylodes macrocephala* Koidz., *Bupleurum falcatum* L., *Codonopsis pilosula* Nannf., *Curcuma longa* L., *Dioscorea polystachya* Turcz., *Glycyrrhiza uralensis* Fisch. ex DC., Ostrea rivularis Gould, *Salvia miltiorrhiza* Bunge, Wolfiporia extensa |
| Li et al. (2017) [53] | Baoyuan decoction and Xiaoyao powder  (保元湯合逍遥散方加減) | *Angelica gigas* Nakai, *Artemisia capillaris* Thunb., *Astragalus propinquus* Schischk., *Atractylodes macrocephala* Koidz., *Bupleurum falcatum* L., *Cinnamomum verum* J.Presl, *Curcuma longa* L., *Glycyrrhiza uralensis* Fisch. ex DC., *Oldenlandia diffusa* Roxb., *Paeonia lactiflora* Pall., *Panax ginseng* C.A mey., *Pulsatilla koreana* Y.N.Lee, Wolfiporia extensa |
| Liu et al. (2017) [54] | Yipi Yanggan decoction  (益脾养肝方) | *Atractylodes macrocephala* Koidz., *Bupleurum falcatum* L., *Cirtus unshiu* Marcow., *Codonopsis pilosula* Nannf., *Curcuma longa* L., *Glycyrrhiza uralensis* Fisch. ex DC., *Oldenlandia diffusa* Roxb., *Poncirus trifoliata* Raf., *Salvia miltiorrhiza* Bunge, *Scutellaria barbata* D.Don, Wolfiporia extensa |
| Pan et al. (2017) [55] | Shentao Ruangan tablet  (参桃软肝方) | *Agrimonia pilosa* Ledeb., Bos taurus Linne var domesticus Gmelin, Panax ginseng C.A mey., *Prunus persica* Batsch, *Pseudostellaria heterophylla* Pax, *Rheum rhabarbarum* L., *Salvia miltiorrhiza* Bunge |
| Song et al. (2017) [56] | Wenyang Jiedu formula  (温阳解毒汤) | *Astragalus propinquus* Schischk., *Myristica fragrans* Houtt., *Rehmannia glutinosa* DC., *Scutellaria baicalensis* Georgi, *Scutellaria barbata* D.Don |
| Wu Mei et al. (2017) [57] | Xiaoliu powder  (消瘤散) | *Bupleurum falcatum* L., *Curcuma longa* L., *Paeonia lactiflora* Pall., Pelodiscus maackii, Pheretima communissima, *Prunus persica* Batsch, Wolfiporia extensa |
| Wu Yunan et al. (2017) [58] | Bielong Ruangan decoction  (鳖龙软肝汤) | *Agrimonia pilosa* Ledeb., *Amana edulis* Honda, *Aralia elata* Seem., *Arisaema amurense* Maxim., *Atractylodes macrocephala* Koidz., Chinemys reevesii, *Coix lacryma-jobi* L., *Echinops latifolius* Tausch, *Eupolyphaga sinensis* Walker, *Impatiens balsamina* L., Pelodiscus maackii, *Poncirus trifoliata* Raf., *Pseudostellaria heterophylla* Pax, *Salvia japonica* Thunb., *Selaginella tamariscina* Spring |
| Xiao et al. (2018) [59] | Jiedu granule  (解毒颗粒) | *Actinidia Valvata* Dunn, *Amana edulis* Honda, Galli Stomachichum Corium, *Salvia japonica* Thunb. |
| Cui et al. (2019) [60] | Herbal decoction^*^ | *Amomum villosum* Lour., *Angelica gigas* Nakai, *Bupleurum falcatum* L., *Coix lacryma-jobi* L., *Crataegus pinnatifida* Bunge, *Curcuma longa* L., *Cuscuta japonica* Choisy, *Eclipta prostrata* L., *Inula helenium* L., *Ligustrum japonicum* Thunb., *Lycium chinense* Mill., *Melia azedarach* L., *Paeonia × suffruticosa* Andrews, *Paeonia lactiflora* Pall., *Panax ginseng* C.A mey., *Pinellia* Ten., *Psoralea corylifolia* L., *Rehmannia glutinosa* DC., *Salvia miltiorrhiza* Bunge, Wolfiporia extensa |
| Yang et al. (2021) [61] | Fuzheng Jiedu Xiaoji formula  (扶正解毒消积方) | *Angelica gigas* Nakai, *Astragalus propinquus* Schischk., *Atractylodes macrocephala* Koidz., *Codonopsis* lanceolate, *Codonopsis pilosula* Nannf., *Curcuma* zedoaria, *Liriope muscari* L.H.Bailey*, Paris verticillata* M.Bieb, *Pinellia* Ten., Wolfiporia extensa |

^*^ The case where only the composition was presented without the specific name of the herbal medicine is indicated
