# Supplementary material for: Adjuvant effect of herbal medicine on transarterial chemoembolization in patients with hepatocellular carcinoma: A systematic review and meta-analysis
Source: Front Oncol. 2023 Feb 9;13:1106827. doi: 10.3389/fonc.2023.1106827 (PMC9948036; doi:10.3389/fonc.2023.1106827)
Supplement: Supplementary file 2 [file Table_2.docx]

**Supplementary Table 2. Frequently used herbs (more than 5 times)**

| **Herb** | **Chinese name** | **Frequency** |
| --- | --- | --- |
| *Atractylodes macrocephala* Koidz. | 白术 | 17 |
| Wolfiporia extensa | 茯苓 | 15 |
| *Curcuma* *longa* L. | 姜黄 | 12 |
| *Bupleurum falcatum* L. | 柴胡 | 12 |
| *Astragalus propinquus* Schischk. | 黄芪 | 11 |
| *Codonopsis pilosula* Nannf. | 党蔘 | 10 |
| *Paeonia lactiflora* Pall. | 勺药 | 10 |
| *Glycyrrhiza uralensis* Fisch. ex DC. | 甘草 | 9 |
| *Oldenlandia diffusa* Roxb. | 百花蛇舌草 | 8 |
| *Salvia miltiorrhiza* Bunge | 丹蔘 | 7 |
| *Pinellia* Ten. | 半夏 | 7 |
| *Panax ginseng* C.A mey. | 人蔘 | 7 |
| *Cirtus unshiu* Marcow. | 陈皮 | 7 |
| *Angelica gigas* Nakai | 当归 | 7 |
| *Crataegus pinnatifida* Bunge | 山查 | 6 |
| *Scutellaria barbata* D.Don | 半枝莲 | 5 |
| *Rehmannia glutinosa* DC. | 地黄 | 5 |
| *Pseudostellaria heterophylla* Pax | 太子参 | 5 |
| *Prunus persica* Batsch | 桃仁 | 5 |
| *Inula helenium* L. | 木香 | 5 |
| Galli Stomachichum Corium | 鸡内金 | 5 |
| *Coix lacryma-jobi* L. | 薏苡仁 | 5 |
| *Agrimonia pilosa* Ledeb. | 仙鹤草 | 5 |
|  | |  |
